# Suboptimal Caloric and Micronutrient Intakes in Female Student Athletes Across Several Division 1 Collegiate Sports

**DOI:** 10.3390/nu17223625

**Published:** 2025-11-20

**Authors:** Rachel L. Victor, Morgan M. Nishisaka, Alexandra F. McGrath, Mia K. Gladding, Liel Grosskopf, Hilla Ben-Moshe, Angelos K. Sikalidis, Aleksandra S. Kristo, Scott K. Reaves

**Affiliations:** 1 Nutrition Program, Department of Food Science and Nutrition, California Polytechnic State University, San Luis Obispo, CA 93407, USA; rlvictor@calpoly.edu (R.L.V.); mnishisa@calpoly.edu (M.M.N.); afmcgrat@calpoly.edu (A.F.M.); mgladdin@calpoly.edu (M.K.G.); lgrossko@calpoly.edu (L.G.); hbenmosh@calpoly.edu (H.B.-M.); asikalid@calpoly.edu (A.K.S.); akristo@calpoly.edu (A.S.K.); 2 Sports Nutrition Program, Department of Athletics, California Polytechnic State University, San Luis Obispo, CA 93407, USA; 3 Cal Poly Personalized Nutrition Research Group, California Polytechnic State University, San Luis Obispo, CA 93407, USA

**Keywords:** female collegiate athletes, micronutrients, athletic performance, relative energy deficiency

## Abstract

Optimal nutrition in athletes can contribute to training adaptations, recovery, performance, and injury prevention in addition to supporting their overall health and well-being. Macronutrient intake and the link to role in athletic performance has been investigated by several studies, while micronutrient intake in athletes appears to be understudied. Therefore, the purpose of this study was to assess caloric and selected micronutrient intakes in female collegiate student-athletes across several sports. The participants from seven sports were instructed to complete 3-day food records; dietary intakes were compared to their individual nutritional needs. Results of this study indicated that average intakes of our participants for vitamin C (132.3%), vitamin K (110.5%), and sodium (173%) intakes were significantly above the recommended intakes. However, average intakes for calories (78.0%), calcium (63.1%), magnesium (68.7%), potassium (83.8%), and iron (80.8%) were all significantly below the recommended intakes. More specifically, indoor volleyball and golf athletes exhibited significantly lower vitamin A intake (53.3% and 43.6%, respectively), while iron insufficiency (% of recommendation) was more pronounced in the indoor volleyball (59.8%), golf (65.4%), and tennis (69.9%) teams. Chronic underconsumption of calories and micronutrients can lead to numerous health and athletic performance related consequences. Thus, recognizing and addressing inadequate intakes is imperative to help the student-athletes meet their needs in alignment with dietary guidelines through nutrition education and counseling, as well as dedicated funding and resources towards promoting their overall health, well-being, and athletic as well as academic performance and success.

## 1. Introduction

Adequate nutritional intake is imperative for supporting athletes’ training adaptation, recovery, performance, injury prevention as well as overall health and well-being [1]. Our work presented herein focused on assessing energy and selected micronutrient intake in female college athletes of various teams, a relatively understudied topic. For certain athletes consuming adequate amounts of food to meet their increased energy demands due to significant training and competition may be challenging, especially when aiming for an increase in body weight and muscle mass [2]. Research suggests that it may be common for college student athletes specifically to not meet dietary recommendations [3,4] with inadequate intake potentially leading to negative consequences for overall health and athletic performance as well as academic performance. Specifically, Kwon et al. recently reported lower than adequate intake of calories and protein in male and female collegiate soccer players [4]. Moreover, Nishisaka et al., showed inadequate energy, protein, and carbohydrate intake relative to recommendations for exercise in individuals in this case Collegiate male basketball players [5]. Importantly the International Olympic Committee in a consensus statement issued on the relative energy deficiency in sports, highlighted the importance of adequate calorie and nutrient intake in both male and female athletes, indicating that widespread deficiencies have been reported thus raising concerns about the health and well-being of athletes including Collegiate athletes [6]. While energy and macronutrient intakes are discussed, little mention and emphasis is given to micronutrients.

However, the fact that an international official athletic entity globally respected raises the issue in a formal manner indicates the importance of the issue and how timely it is. The purpose of this study was to assess the caloric and selected micronutrient intakes of NCAA Division I female student-athletes and compare them to dietary recommendations. While energy intake is important and was also assessed, our key focus was on those micronutrients, which are particularly important for athletic performance, and have also been thought to potentially be taken at lower than optimal amounts.

Recognizing nutrient deficiencies can aid in undertaking preventative efforts and providing appropriate nutrition counseling and resources to athletes. Many collegiate athletes report that they lack the time, funding, cooking skills, and resources it takes to meet their nutritional needs [7,8]. Some research suggests that food insecurity affects up to 65% of student-athletes at some universities [9,10]. One study assessed 24 h recalls and food frequency-questionnaires and found that only 9% of NCAA female athletes met their total energy needs [11].

The balance of energy intake and energy expenditure can play a critical role in athletic performance, body composition, training adaptations, and injury prevention [12]. Chronic underconsumption of calories can lead to low energy, impaired metabolic rate, hormonal disruptions, menstrual dysfunction, and reduced bone health, immunity, protein synthesis, and impaired cardiovascular health [1]. Relative energy deficiency in sports (RED-s) is a condition of chronic low energy consumption that does not meet energy demands. Whether the underconsumption is intentional or not, RED-s can have long-term consequences on health and sport performance [13].

Our study specifically assessed the daily intake of calories, vitamin A, vitamin B_12_, vitamin C, vitamin K, folate, calcium, iron, magnesium, potassium, and sodium in female student-athletes. The 3-day average intakes relative to individual needs were explored for all participants, within each sports team, and between teams. The micronutrients assessed were selected for assessment due to their functions in athletic training and performance.

More specifically, vitamin A is key for optimal vision, bone health, and skin vigor, vitamin B_12_ is critical for red blood cell production and energy metabolism. Vitamin C supports the immune system, extends antioxidant defense and benefits in post-exercise recovery. Vitamin K is crucial for optimal blood clotting and bone density regulation. Folate is important for cell division and DNA synthesis thus becomes important in the context of muscle turnover, bone remodeling, and strength and resistance exercise. Sodium optimizes fluid balance and nerve function during exercise. Calcium is key for bone strength and muscle contraction. Iron is critical for oxygen transport and energy metabolism. Magnesium plays a role in muscle function and is a cofactor participating in energy production. Potassium is essential for muscle contraction and fluid balance as it contributes to preventing cramping and supports hydration status. Therefore, all these nutrients are crucial for maximizing athletic performance and recovery.

The main driver of our study herein was to obtain data on the degree to which female college athletes meet required levels of caloric and micronutrient intake through their diet. Given that our population is still in a developmental phase, combined with the increased needs due to academic and athletic performance, it becomes critical to have adequate nutrition qualitatively and quantitatively to remain healthy and maximize performance.

All participants in our study were student athletes (college students who participate in the collegiate teams pertinent to their sport). The level is semi-professional while highly structured and rigorous with arranged training scheduled daily and regular weekly competitive games during the season, while also quite demanding when considering age, athletic and academic obligations of college student athletes. This is a condition somewhat unique and idiosyncratic in American Higher Education in terms of college sports, which often function as feeders for the professional leagues/teams and sometimes even Olympic teams.

Given field perception and the limited literature available we hypothesized that our study population would be deficient in caloric and micronutrient intake assessed. Our study aimed to discern that, quantify it, and use this data for future reference and to inform strategies towards improving nutritional support for college student athletes on campuses. More specifically, our study can update programs on nutrition education and consultation as well as sports nutrition initiatives that can improve nutrition status of college female athletes and subsequently contribute to improving their athletic and academic performance as well as their overall health.

## 2. Materials and Methods

### 2.1. Participants

Participants were from female Athletics teams of California Polytechnic State University. Inclusion criteria were over 18 years old, female, not pregnant, non-smoker, cleared by the Sports Medicine Physician to compete, generally healthy with no medical conditions that affect nutrient requirements. Participants were excluded if their food records did not include all 3 days, if they did not provide sufficient information to accurately report their intake. All athletes in the University Athletics Teams (~550 athletes participate in teams each year) were approached over a six-year period with the opportunity to voluntarily participate in our Sports Nutrition research study. All participants provided written consent (IRB No. 2018-274) prior to their participation and were informed of the procedures, benefits, and risks of their involvement in the research as described previously [4]. A total of 400 NCAA Division I student athletes voluntarily submitted 3-day food records and were evaluated for eligibility for the study based on inclusion and exclusion criteria. Ultimately, we were able to gather complete 3-day food records from 149 female student-athletes between 2017 and 2023, who all met inclusion criteria. The study participants were members of one of the following women’s teams: beach volleyball (*n* = 28), indoor volleyball (*n* = 16), swim and dive (*n* = 19), track and field (*n* = 25), golf (*n* = 9), soccer (*n* = 44), or tennis (*n* = 8). Descriptive characteristics of the participants are summarized in Table 1. These teams were selected because the number of participants to be analyzed reflected a significant proportion (>50%) of the typical roster of the respective female team members. The study was of an exploratory nature and aimed to assess how the caloric and micronutrient intakes of college student athletes compared to optimal recommended amounts as determined in a personalized manner through the ESHA platform.

### 2.2. Data Collection

As previously reported [4], the participants voluntarily submitted standardized 3-day food record forms that included verbal and written instructions to accurately record all foods and beverages consumed. Accepted food records included those with details about the type, amount, and method of preparation for each food or beverage consumed. The participants were asked to include two weekdays and one weekend day in their food diaries to better depict any likely changes in dietary habits throughout the week. The student athletes included their self-reported height, weight, and age in their food records. If the participant submitted multiple food records, only their first food record was included. Any subsequent food records from future dates were not included. Trained sports nutrition research assistants manually entered and analyzed data from food diaries using the Food Processor III Nutrition Analysis Software (version 11.11.32, ESHA Nutrition Research, Salem, OR, USA). Analysis assessed the daily calorie, vitamin A, vitamin B_12_, vitamin C, vitamin K, folate, calcium, iron, magnesium, potassium, and sodium intakes of female student-athletes. These vitamins and minerals were chosen for assessment because of their functions in overall health and athletic training and performance. Energy and micronutrient needs/recommendations were determined individually for each participant based on their height, weight, age, sex, and activity level, using the ESHA platform. To assess actual energy and micronutrient intakes, three-day averages of total intakes for calories and for each of the selected micronutrients were calculated for each participant.

### 2.3. Analyses of Δ-Intake of All Athletes for Calories and Each Nutrient

This study assessed the average daily calorie, vitamin A, vitamin B_12_, vitamin C, vitamin K, folate, calcium, iron, magnesium, potassium, and sodium intake values of each female student-athlete and compared those to respective individualized Dietary Reference Intakes (DRIs). The DRI values used for each of these nutrients were from the most recent published reports issued by the Food and Nutrition Board of the National Academies of Sciences, Engineering, and Medicine. The DRIs for the general population were used for the purpose of our study. In any event there are no DRIs for athletes and while there are other types of recommendations for athletes since our participants while student athletes cannot be classified as “athletes” as they do not compete at the professional level, it would not be sound to use any other recommendation other than the DRIs. The DRI reports are available at www.nap.edu. The reports present Recommended Dietary Allowances (RDAs) and Adequate Intakes (AIs) for essential vitamins and elements (minerals). An RDA is the average daily dietary intake level sufficient to meet the nutrient requirements of nearly all (97–98 percent) healthy individuals in a group. It is calculated from an Estimated Average Requirement (EAR). If sufficient scientific evidence is not available to establish an EAR, and thus calculate an RDA, an AI is recommended. DRI committees for each nutrient periodically meet to address the status of data related to each nutrient and whether changes to the current DRI are warranted. Reports that contain the most recent DRI changes, even if the report was published a few years ago, are considered to be the nutrient’s current recommended intake. Sources that contain nutrients examined in our study include: Dietary Reference Intakes for Calcium, Phosphorous, Magnesium, Vitamin D, and Fluoride (1997); Dietary Reference Intakes for Thiamin, Riboflavin, Niacin, Vitamin B_6_, Folate, Vitamin B_12_, Pantothenic Acid, Biotin, and Choline (1998); Dietary Reference Intakes for Vitamin C, Vitamin E, Selenium, and Carotenoids (2000); Dietary Reference Intakes for Vitamin A, Vitamin K, Arsenic, Boron, Chromium, Copper, Iodine, Iron, Manganese, Molybdenum, Nickel, Silicon, Vanadium, and Zinc (2001); Dietary Reference Intakes for Calcium and Vitamin D (2011); and Dietary Reference Intakes for Sodium and Potassium (2019). These reports may be accessed via www.nap.edu.

For Table 2, the difference between the intake value and the respective DRI value was calculated and then expressed as Δ-intake. This Δ-intake value constitutes a measure by which the extent to which the DRI is met could be easily assessed. Specifically, meeting the DRI produces Δ-intake = 0, exceeding the DRI produces Δ-intake > 0, and being below the DRI produces Δ-intake < 0. The averages of the Δ-intakes of all athletes were produced to indicate the overall status of the study population in reference to recommended intakes for calories and each micronutrient (data: Table 2).

### 2.4. Dietary Intakes Relative to DRI Recommendations Within Teams

For each team, average actual intakes for calories and each nutrient were calculated and were reported as compared to the DRI for each nutrient. It should be noted, our population (student athletes) is idiosyncratic, as a group may span two different age-specific recommendations for some nutrients. We therefore expressed all data as a percentage relative to individualized energy and nutrient recommendations so that if an athlete consumed a nutrient at the recommended level it was expressed as 100%. This approach was taken in order to assess the extent to which the entire population of our study met DRIs for calories and for each micronutrient (data: Table 3, Table 4 and Table 5).

### 2.5. Statistical Analyses

Statistical analyses were conducted using JMP Pro software (version 16.0; SAS Institute Inc. (Cary, NC, USA); JMP Statistical Discovery, LLC, Cary, NC, USA). For ∆-intakes of all athletes (Table 2), one sided *t*-tests were conducted to test whether the difference in average reported intakes and the average recommended intakes (∆-intakes) were different from 0 for caloric intake and each micronutrient intake for all participants and within teams. “Different from 0” is referring to the recommended intake as “0”. Therefore, any value different from 0 indicates that the athlete’s average intake was different from the recommended intake. Bonferroni adjustments were applied to the analyses to control for type I errors, resulting in the establishment of a significance threshold of alpha < 0.0045. A one-way ANOVA test was administered to examine any differences between teams in BMI. Data are presented as means ± standard deviation (SD) or percentages. For comparisons of calorie, vitamin and mineral intakes vs. recommendations, one-sided *t*-tests were conducted to test whether the difference in average reported intakes and the average recommended intakes were significant.

Normality of differences between observed and recommended intakes was assessed using the Shapiro–Wilk test. For nutrients where this assumption was violated, Wilcoxon Signed-Rank tests were used as nonparametric alternatives to one-sample *t*-tests to determine whether the median intake differed from the recommended nutrient intake or to express a descriptive statistic.

## 3. Results

### 3.1. BMI

A one-way ANOVA test was administered to examine any differences between teams in BMI values. No significant differences were seen in BMI among teams.

### 3.2. ∆-Intakes of All Athletes

Normality of differences between observed and recommended intakes was assessed using the Shapiro–Wilk test. For all ∆-intake calorie and nutrients data this normality assumption was violated, and Wilcoxon Signed-Rank tests were used to compare calories and micronutrient actual intakes versus individualized DRIs for all athletes. We also compared median values for intake vs. median values recommendations. The results are summarized in Table 2. We did not find significant differences in intakes compared to DRIs for vitamin A, vitamin B_12_ and folate. We found significant evidence that suggests the average intakes were below the average recommended intakes for calories (*p* < 0.0001), calcium (*p* < 0.0001), iron (*p* < 0.0001), magnesium (*p* < 0.0001), and potassium (*p* < 0.0006). We found significant evidence that suggests the average intakes were above the average recommended intakes for vitamin C (*p* < 0.0001), vitamin K (*p* < 0.0021), and sodium (*p* < 0.0001).

### 3.3. Dietary Intakes Relative to Dietary Reference Intakes Recommendations Within Teams

For data that followed normal distribution, one sided *t*-tests were also conducted for the intakes relative to DRI recommendations within teams. Intakes vs. DRI recommendations by team are summarized in Table 3, Table 4 and Table 5. The following results are presented by calories (Table 3), each vitamin (Table 4) and each mineral (Table 5). For data in which the normality assumption was violated, the Wilcoxon Signed-Rank tests were used.

#### 3.3.1. Calories

We did not find significant evidence that suggests the average intakes for calories were different from the average recommended intakes for the athletes on the Tennis team, although when actual intake data was expressed as percent of recommended intake the divergence was significant. We found significant evidence that suggests the average intakes for calories were below the average recommended intakes for the athletes on the Beach Volleyball, Indoor Volleyball, Swim and Dive, Track and Field, Golf and Soccer teams. These results are summarized in Table 3.

#### 3.3.2. Vitamin A

We did not find significant evidence that suggests the average intakes for vitamin A were different from average recommended intakes for the athletes on the Beach Volleyball, Swim and Dive, Track and Field, Soccer, or Tennis teams. We found significant evidence that suggests average intakes for vitamin A were below average recommended intakes for athletes on the Indoor Volleyball and Golf teams. These results are summarized in Table 4.

#### 3.3.3. Vitamin B_12_

We did not find significant evidence that suggests average intakes for vitamin B_12_ were different from the average recommended intakes with nearly every team with the exception being Soccer. When data were expressed as a percentage relative to the DRI recommendation the value of 1.7 µg was significantly less than the 2.4 µg recommendation. These results are summarized in Table 4.

#### 3.3.4. Vitamin C

We did not find significant evidence that suggests average intakes for vitamin C were different from average recommended intakes within the athletes on the Indoor Volleyball, Track, Swim and Dive, Track and Field, Golf, or Tennis teams. We found significant evidence that suggests average intakes for vitamin C were above the average recommended intakes for athletes on the Beach Volleyball and Soccer teams. These results are summarized in Table 4.

#### 3.3.5. Vitamin K

We did not find significant evidence that suggests average intakes for vitamin K were different from average recommended intakes within the athletes on the Beach Volleyball, Indoor Volleyball, Swim and Dive, Track and Field, Golf, or Tennis teams. We found evidence that the average intakes for vitamin K were above average recommended intakes for athletes on the Soccer team. These results are summarized in Table 4.

#### 3.3.6. Folate

We did not find significant evidence that suggests average intakes for folate were different from average recommended intakes within the athletes on the Beach Volleyball, Swim and Dive, Track and Field teams. We found significant evidence that suggests the average intakes for folate were different from the average recommended intakes for athletes on the Indoor Volleyball, Soccer, Golf, and Tennis teams. The evidence suggests that Soccer was the only team with intake significantly above the recommended amount. These results are summarized in Table 4.

#### 3.3.7. Calcium

We found evidence that the intakes for calcium were significantly below the recommended intakes for all of the teams. These results are summarized in Table 5.

#### 3.3.8. Iron

We did not find significant evidence that suggests average intakes for iron were different from average recommended intakes within the athletes on the Beach Volleyball, Track and Field teams. We found significant evidence that suggests average intakes for iron were below average recommended intakes for athletes on the Indoor Volleyball, Swim and Dive, Soccer, Golf, and Tennis teams. These results are summarized in Table 5.

#### 3.3.9. Magnesium

We did not find significant evidence that suggests average intakes for magnesium were different from average recommended intakes within the athletes on the Indoor Volleyball, Beach Volleyball, and Golf teams. We found significant evidence that suggests average intakes for magnesium were below the average recommended intakes for athletes on the Swim and Dive, Track and Field, Soccer, and Tennis teams. These results are summarized in Table 5.

#### 3.3.10. Potassium

We did not find significant evidence that suggests average intakes for potassium were different from average recommended intakes within the athletes on the Beach Volleyball, Track and Field, and Soccer teams. We found significant evidence that suggests average intakes for potassium were below average recommended intakes for athletes on the Indoor Volleyball, Swim and Dive, Golf and Tennis teams. These results are summarized in Table 5.

#### 3.3.11. Sodium

We found significant evidence that suggests average intakes for sodium were above average recommended intakes for all teams. These results are summarized in Table 5.

## 4. Discussion

The purpose of this study was to assess female student-athletes’ caloric and selected micronutrient intakes compared to their individualized recommended intakes. To the best of our knowledge, our study is unique given the studied population, collection of sports, and the individualized assessments conducted, as well as the combination of nutrients assessed. We chose to investigate these variables given our experiences and the literature that these nutrients can be challenging for female athletes to obtain in adequate amounts. Adequate caloric and micronutrient intakes are essential for general health as well as for athletic performance, training, and recovery, while several factors contribute to success in athletic performance with diet playing a critical role [14].

### 4.1. Importance of Selected Assessed Micronutrients

We decided to select the micronutrients mentioned for assessment given their importance in terms of optimization for athletic performance. Moreover, the assessment of these micronutrients is understudied especially in this population as the focus is primarily on energy and macronutrient intake. Therefore, our study is contributing uniquely in terms of assessing and understanding the nutritional needs of female college athletes. The importance of selected micronutrients becomes clear when considering how deficiencies can negatively impact athletic performance.

More specifically, calcium is a major player in muscle contraction, nerve signaling, and bone mineralization. Suboptimal calcium levels can lead to muscle cramps, spasms, and weakness, thus compromising athletic performance. Moreover, prolonged calcium deficiency can impair bone density, increasing the risk of fractures and conditions such as osteopenia or osteoporosis, especially later in life. This is particularly concerning for athletes who engage in high-impact activities or those in weight-bearing sports, since they place greater stress on their bones.

Iron and magnesium deficiencies also extend negative impact on athletic performance as well as bone health. Iron is critical for hemoglobin production. Low iron status leads to anemia, in turn resulting in fatigue, diminished endurance, and slower recovery times in athletes. This becomes even more challenging in women athletes. Magnesium is involved in several metabolic processes such as energy production, muscle function, and bone mineralization. Magnesium deficiency can cause muscle cramps, fatigue, and increase injury susceptibility. Furthermore, magnesium contributes to establishing optimal calcium balance in the skeleton, hence magnesium deficiency can indirectly contribute to weaker bones and thus increase risk for injuries. Therefore, deficiencies of the micronutrients discussed especially when they occur collectively severely undermine muscle performance and skeletal integrity, diminishing an athlete’s capacity to train, perform, and recover effectively.

### 4.2. Intakes Above Recommendations

Our results indicated that the average intakes of all participants were above the average recommended intakes for vitamin C by 32.25%, vitamin K by 10.53%, and sodium by 72.96%. Also, the average vitamin C intake for Beach Volleyball and Soccer exceeded their average recommended intakes by 66.1% and 57.1%, respectively. The average vitamin C intakes for all participants and within teams were below the UL of 2000 mg/day. Interestingly, Soccer was the only team that significantly over-consumed vitamin K exceeding recommendations by 29.5%, but with no UL established for vitamin K it does not represent a point of concern.

For sodium, within teams, significant evidence suggests the following teams’ average consumption exceeded that of the recommended intake: Beach Volleyball by 91.9%, Indoor Volleyball by 57.2%, Swim and Dive by 70.0%, Track and Field by 102.3%, Golf by 59.6%, Soccer by 78.6% and Tennis by 104.8%. The participants’ average sodium intake exceeded the UL of 2300 mg/day by 13.0%. While sodium intakes that exceed UL may be associated with an increased risk of kidney disease, osteoporosis, high blood pressure, and cardiovascular disease [15], our findings do not raise particular concern, given the magnitude observed and the fact that the participants are athletes. In fact, some athletes, depending on the sport, may have an increased requirement for sodium to replace the sodium lost in sweat.

### 4.3. Intakes Below Recommendations

We found that the average intakes of all participants were below the average recommended intakes for calories by 22%. Values for specific teams that were below the recommended calories were as follows: Beach Volleyball by 19.8%, Indoor Volleyball by 31.5%, Swim and Dive by 18.7%, Track and Field by 19.1%, Golf by 33.4%, Soccer by 25.6%, and Tennis 9.5%. Several health and athletic related consequences are associated with the underconsumption of calories. Research suggests that female athletes have a higher risk of experiencing RED-s compared to male athletes [16,17,18,19]. Athletes of endurance, aesthetic, and weight-class sports also have a higher risk of experiencing RED-s [1]. It is important to recognize and address signs of RED-s before long-term consequences emerge.

Given the apparent inadequacy in calorie intake, it was not surprising that intake levels of some of the micronutrients we studied were below recommendations. For example, the average intakes of all participants were below the average recommended intakes for calcium by 36.9%. Analysis by teams indicated the following teams’ average calcium intakes were significantly below their average recommended intakes: Beach Volleyball by 22.2%, Indoor Volleyball by 32.1%, Swim and Dive by 31.0%, Track and Field by 38.6%, Golf by 48.5%, Soccer by 36.8%, and Tennis by 35.5%. Our data tend to align with data from several other studies on female collegiate athletes that reported less than adequate calcium intake levels of 876.7 mg/day in soccer players [20], 726.6 mg/day in basketball players [21], and 759.7 mg/day in volleyball players [21]. However, one study reported calcium intake of 1131 mg/day and this amount exceeds the recommended amount [22]. The aforementioned study by Petersen et al., was in swimmers and divers and presents an interesting comparison with swimmers and divers of our study that had a calcium intake of 582 mg/day. It is unclear why calcium intake levels in the Peterson et al. study appear to be higher than many other studies including ours, as they also conducted 3-day food records and similarly reported calorie intake (i.e., 2405 kcals/day) comparable to the other studies cited above. Petersen et al. did use a different nutrient database, and it was performed nearly 20 years ago, so perhaps these factors play at least some type of role in the variation.

There appears to be several studies reporting lower than adequate calcium intake in female collegiate athletes to warrant concern. Athletes participating in high-impact sports that require running and jumping, such as basketball, volleyball, track and field, and gymnastics have an increased risk of developing stress fractures [23]. The impact these athletes experience, in combination with low calcium intake, can increase their risk of stress fractures [24]. The risk can be further exacerbated in athletes experiencing RED-s. Low calcium intake during early adulthood is also associated with an increased risk of osteoporosis for postmenopausal females [25].

We also evaluated iron intake and found significant evidence that indicates the average intakes of all participants were below the average recommended intakes for iron by 19.2% (approximately 12 mg/day). We found significant evidence that suggests the following teams’ average iron intakes fell below their average recommended intakes: Indoor Volleyball by 40.2%, Golf by 34.6%, and Tennis by 30.1%. A survey of the literature in female collegiate athletes suggests iron inadequacy is relatively common. For example, intake of iron was determined to be 12.6 mg/day [21], 11.2 mg/day [21], 14.3 mg/day [20], and 16.3 mg/day [22] in a variety of different female collegiate sports teams at different universities. A study by Dunn et al. examined the prevalence of iron deficiency in female collegiate athletes and reported that 57.7% of the 336 participants had ferritin values under 40 ng/mL, the level used in the Division I screening protocol, as the cutoff below which female athletes are to be referred for nutritional counseling. These intake and ferritin findings from other studies support ours and are cause for concern given inadequate iron consumption can compromise overall health and athletic performance. Chronic iron under consumption causes a cascade of events involving reduced iron storage, uptake, and transport that can eventually lead to iron deficiency anemia [26]. Iron deficiency anemia can severely hinder athletic performance, which can also compromise cognitive function, which is also important in athletic performance [27]. Females have a higher risk of iron deficiency compared to males thereby emphasizing the need to assess and address poor iron status of female athletes.

We found significant evidence that suggests the average intakes of all participants were below the average recommended intakes for magnesium by 31.3% and this translates to consumption of 213 mg/day vs. the 310 mg/day recommended amount. Reports from other studies included intakes of 246 mg/day [22], 165 mg/day [21], and 242 mg/day [20]. Consistency of lower than recommended levels in all the studies mentioned suggests a pattern of insufficient magnesium in female collegiate athletes.

Chronic inadequate magnesium intake can lead to impaired magnesium balance. This may be associated with skeletal deformities, cardiovascular diseases, and metabolic syndrome [28]. Magnesium’s roles in glycolysis, ATP synthesis and utilization, and bone integrity make it an essential nutrient of added value for athletic performance. Therefore, inadequate availability can slow down or limit these processes or utilizations and can ultimately cause fatigue and muscle weakness [29,30]. Magnesium deficiency is associated with reduced bone stiffness, increased osteoclast activity, and decreased osteoblast activity [31,32]. Research also suggests that magnesium needs may be increased in athletes because there are increased magnesium losses during exercise from sweat, urine, and alterations in blood magnesium levels [29].

We also presented evidence that suggests the average intakes of all participants were below the average recommended intakes for potassium by 16.2% and this translates to consumption of approximately 1928–2179 mg/day. The potassium DRIs (Adequate Intake) are different for 18–19-year-olds (2300 mg) and 19+ year olds (2600 mg). Other studies have also reported intake values for potassium including 2422 mg/day [20], 1877 mg/day [21], and 2547 mg/day [22]. Inadequate potassium consumption is associated with compromised health and athletic performance. Some of these consequences include increased blood pressure, kidney stone risk, bone turnover, and urinary calcium excretion. Potassium’s roles in muscle contraction, insulin secretion, bone health, and electrolyte balance make it an imperative nutrient for athletes to consume adequate amounts of [33].

Within team analyses showed evidence of intakes below recommendations for vitamin A and folate that total participant analyses did not. We found significant results that suggest the average intake of vitamin A was well below the average recommended intake for Indoor Volleyball team and the Golf team in which they consumed approximately half the recommended amount. Vitamin A status was reported by Shoemaker et al. and indicated lower than adequate intake for Women’s Basketball players but higher than adequate in Volleyball players. Barney et al. (2024) reported vitamin A intake was more than adequate in female cross-country runners [34]. Significant evidence also suggests that average folate intakes were shown to be below recommended intakes for Indoor Volleyball at 56.2%, Golf at 69.4%, and Tennis at 59.5% of recommended intakes in our studies. However, Barney et al., in their study indicated more than adequate folate was consumed by cross country runners. On the other hand, Green et al. (2020) reported that female collegiate gymnasts consumed less than adequate levels of folate overall as assessed at three different time points throughout the year [35]. Given the variability of folate intake in female collegiate athletes reported in studies it appears to be an important nutrient to assess in this population.

### 4.4. Strengths, Limitations, and Future Strategies

The use of ESHA for nutritional analysis was a strength of this study. It is considered a very appropriate tool for nutrition analysis and is used extensively for clinical and research purposes [36]. Another strength of this study was the analysis of the intakes of multiple different teams with athletes of different ages. We were able to assess the intakes of athletes participating in seven different sports ranging from freshmen to seniors. Variability within the study population creates a broader range of athletes to help recognize similarities, trends, and differences between sports and different grade levels. However, self-reporting through a 3-day food record and seasonality may be considered as limitations. The limitations could also include the relatively small sample size or lack of comparison by age group. However, the weekly frequency between sports and athletes could open up a debate on variation in results.

Potentially helpful strategies for collegiate sports nutrition programs can be extended through nutrition education initiatives. Current research suggests that sports nutrition knowledge among NCAA collegiate athletes is poor and that it does not meet current recommendations [11,37,38,39,40]. Research has shown that increasing and bettering nutrition education from qualified nutrition professionals can help improve eating habits to meet needs [41,42,43]. Providing nutrition education through services such as cooking lessons, team nutrition education classes, and one-on-one nutrition education meetings can help the athletes meet their needs [44]. Providing athletes with appropriate snacks and food can be a convenient way to increase their caloric and micronutrient intakes to better meet their needs.

Moreover, personalized nutrition counseling whereby college athletes receive one-on-one sessions with a sports dietitian provides tailored informed advice based on the athlete’s individual needs, training schedules, and goals. Also, hosting workshops and seminars on topics such as pre- and post-workout meals, hydration strategies, and supplements can help by providing strategies and debunking myths thus empowering student athletes and helping them develop skills and make good practical decisions in terms of their diets. Leveraging social media and digital platforms can help promote positive messaging and information on practices and approaches that can help college athletes. Finally, team-based nutrition challenges and peer mentorship programs can increase awareness, build camaraderie and trust, induce self-esteem and positive self-imaging, while also modeling and motivating college athletes to adopt positive dietary behaviors. In addition to healthier eating and nutrition knowledge reinforcement, these approaches can also contribute to the enhancement of team-spirit and accountability.

Other methods to potentially help collegiate athletes meet their nutritional needs can be through providing funding and nutritional resources. Vento et al., 2023 [45] examined how funding and college-provided nutritional resources were associated with diet quality among female collegiate athletes. They assessed funding, college-provided nutritional resources, and diet quality among National Collegiate Athletic Association (NCAA), National Junior College Athletic Association (NJCAA), and club female athletes [45]. They found that the NCAA athletes reported more funding than NJCAA and club athletes. NJCAA athletes reported insufficient funds for purchasing foods and fewer college-provided nutritional resources while also showing lower diet quality scores than NCAA and club athletes. Overall, these findings suggest that funding and nutritional resources may help student-athletes meet their nutritional needs.

## 5. Conclusions

The overall purpose of this study was to assess how female athletes’ intake for calories and selected micronutrients compare to their recommended intakes. We found significant evidence that suggests many of the participants consumed more than the recommendations for vitamin C, vitamin K, and sodium. The average intakes did not exceed the UL for vitamin C or vitamin K; therefore, their consumptions remained at safe levels. The average intakes did exceed the UL for sodium. We also found evidence that suggests many of the participants were receiving fewer calories, and less calcium, iron, magnesium, and potassium than their recommendations. Our results underline potential deficiencies in the nutrition of college female athletes. Recognizing such a possibility and setting systems in place to assess and address when low consumption of calories and micronutrients occurs, is imperative to help student-athletes meet their needs. Understanding what these student-athletes are under consuming can help direct and facilitate nutrition support through nutrition education, individualized nutrition counseling, funding, and nutritional resources. Helping athletes consume adequate calories and nutrients can promote their overall health, well-being, athletic and academic performance.

## Figures and Tables

**Table 1:** Descriptive characteristics of participants ^1^.

Sport	Beach Volleyball (*n* = 28) Indoor Volleyball (*n* = 16) Swim and Dive (*n* = 19) Track and Field (*n* = 25) Golf (*n* = 9) Soccer (*n* = 44) Tennis (*n* = 8)
Age (years)	19.17 ± 1.21
Weight (kg)	64.54 ± 8.90
Height (m)	1.70 ± 0.09
BMI	22.32 ± 2.10

^1^ Data are presented as mean values ± SD.

**Table 2:** ∆-intakes of all athletes ^1^.

	∆-Intakes (Median) + IQR	*p*-Value	Percentage of Recommended Intake (Median + IQR)	*p*-Value
Calories	−569.49 (−937.56–−203.33)	*p* < 0.0001 *	78.01% (64.18–91.28%)	*p* < 0.0001 *
Vitamin A (mg RAE)	−154.92 (−426.63–+191.40)	*p* < 0.0057	77.87% (39.05–127.34%)	*p* < 0.0057
Vitamin B_12_ (µg)	−0.27 (−1.09–+1.16)	*p* < 0.44	88.75% (54.58–148.13%)	*p* < 0.44
Vitamin C (mg)	+23.8 (−17.46–+85.44)	*p* < 0.0001 *	132.25% (75.35–223.65%)	*p* < 0.0001 *
Vitamin K (µg)	+7.9 (−43.41–+79.62)	*p* < 0.0021 *	110.53% (46.04–194.92%)	*p* < 0.0017 *
Folate (µg DFE)	−53.17 (−175.99–+103.76)	*p* < 0.029	86.71% (56.00–125.94%)	*p* < 0.029
Calcium (mg)	−411.15 (−620.42–−182.17)	*p* < 0.0001 *	63.06% (42.48–83.29%)	*p* < 0.0001 *
Iron (mg)	−3.39 (−6.93–+0.37)	*p* < 0.0001 *	80.80% (60.03–102.22%)	*p* < 0.0001 *
Magnesium (mg)	−97.08 (−175.18–−7.3)	*p* < 0.0001 *	68.68% (46.43–97.80%)	*p* < 0.0001 *
Potassium (mg)	−399.8 (−802.61–+393.05)	*p* < 0.0006 *	83.81% (66.57–116.05%)	*p* < 0.0007 *
Sodium (mg)	+1094.47 (+490–+1965.18)	*p* < 0.0001 *	172.96% (132.69–231.01%)	*p* < 0.0001 *

^1^ Data are presented as median values with interquartile ranges (IQR); * Statistically significant ∆-intakes.

**Table 3:** Comparisons of calories consumed vs. caloric recommendations.

Team	Calories
Beach Volleyball	2123.3 ± 503.3 * (80.2% of Rec.) *
Indoor Volleyball	1886.5 ± 477.8 * (68.5% of Rec.) *
Swim and Dive	2064.5 ± 464.8 * (81.3% of Rec.) *
Track and Field	2012.2 ± 444.5 * (80.9% of Rec.) *
Soccer	1878.46 (1596.60–1878.46) (74.4% (64.5–95.1%) of Rec.) *
Golf	1608.8 ± 259.0 * (66.6% of Rec.) *
Tennis	2247.8 ± 600.5 (90.5% of Rec.) *

Note: Data are mean values ± SD; * denotes significant difference between intake and respective recommendation. Soccer data did not fit normality so median, 25% and 75% interquartile ranges are shown.

**Table 4:** Comparisons of amount of vitamins consumed vs. DRI recommendations per respective participant age (18 years of age or >19 years of age).

Team	Vitamin A DRI 700 µg	Vitamin B_12_ DRI 2.4 µg	Vitamin C DRI 65 or 75 mg	Vitamin K DRI 75 or 90 µg	Folate DRI 400 µg DFE
Beach Volleyball	722.98 (485.21–1166.57) (103.3% (69.3–166.7%) of Rec.)	2.85 (1.68–4.52) (118.5% (70.1–188.3%) of Rec.)	112.06 (62.26–155.10) (166.1% (91.7–218.7%) of Rec.) *	114.27 (49.26–176.10) (128.5% (54.7–205.7%) of Rec.)	529.8 ± 300.8 (132.4% of Rec.)
Indoor Volleyball	372.0 ± 358.5 * (53.3% (25.4–72.2%) of Rec.) *	2.2 (0.9–2.7) (91.3% (39.4–113.6%) of Rec.)	102.9 (63.9–192.0) (146.8% (85.3–257.7%) of Rec.)	38.7 (22.5–111.2) (49.3% (25.0–123.5%) of Rec.)	204.3 (166.2–265.5) (56.2% of Rec.) *
Swim and Dive	567.7 ± 414.2 (81.1% of Rec.)	2.5 ± 2.0 (105.2% of Rec.)	86.2 ± 59.8 (120.0% of Rec.)	63.7 (21.0–156.6) (84.3% (23.2–182.6%) of Rec.)	352.0 ± 208.8 (88.0% of Rec.)
Track and Field	684.4 (387.8–955.4) (97.8% (55.4–136.5%) of Rec.)	2.5 (1.5–4.8) (105.0% (64.4–197.9%) of Rec.)	95.5 (49.6–191.7) (127.3% (71.4–272.3%) of Rec.)	107.35 (36.4–182.1) (119.3% (48.5–212.2%) of Rec.)	365.2 (228.4–467.4) (91.3% (57.1–116.9%) of Rec.)
Soccer	566.0 (310.9–880.3) (80.9% (44.4–125.8%) of Rec.)	1.7 (1.1–2.6) (70.2% (43.8–109.5%) of Rec.) *	109.6 ± 55.0 * (157.1% of Rec.) *	114.6 (66.7–168.6) (129.5% (78.3–212.7) of Rec.) *	428.7 (270.0–537.5) * (107.2% (67.5–134.4%) of Rec.)
Golf	305.3 (240.0–513.8) (43.6% (34.3–73.5%) of Rec.) *	2.6 ± 1.3 (107.1% of Rec.)	48.9 (33.3–135.8) (65.2% (51.2–181.0%) of Rec.)	87.4 ± 61.3 (100.4% of Rec.)	277.4 ± 91.8 * (69.4% of Rec.) *
Tennis	570.1 ± 577.0 (81.4% of Rec.)	2.0 ± 2.3 (85.2% of Rec.)	89.5 (19.8–126.7) (119.3% (26.5–189.2%) of Rec.)	120.2 ± 98.2 (137.8% of Rec.)	238.0 ± 105.7 * (59.5% of Rec.) *

Note: Data are mean values ± SD or median values with 25% and 75% interquartile ranges; * denotes significant difference between intake and Dietary Reference Intake (DRI) recommendation.

**Table 5:** Comparisons of amount of minerals consumed vs. DRI recommendations per respective participant age (18 years of age or >19 years of age).

Team	Calcium DRI 1300 or 1000 mg	Iron DRI 15 or 18 mg	Magnesium DRI 360 or 310 mg	Potassium DRI 2300 or 2600 mg	Sodium DRI 1500 mg
Beach Volleyball	859.4 ± 335.2 * (77.8% of Rec.) *	17.8 ± 7.4 (108.1% of Rec.)	312.5 ± 139.6 (94.8% of Rec.)	2686.1 ± 824.7 (109.3% of Rec.)	2878.1 ± 1121.2 * (191.9% of Rec.)
Indoor Volleyball	601.6 ± 258.2 * (57.9% of Rec.) *	10.2 (8.8–15.0) (59.8% (54.2–83.5%) of Rec.) *	184.0 (151.6–278.1) (59.4% (44.7–89.7%) of Rec.)	1717.3 (1308.0–2101.7) (69.9% (53.6–80.8%) of Rec.) *	2357.9 (2027.0–3415.4) * (157.2% (135.1–227.7%) of Rec.) *
Swim and Dive	655.6 ± 236.5 * (59.0% of Rec.) *	12.9 ± 4.8 * (78.2% of Rec.) *	189.9 (133.8–213.5) (58.2% (41.1–65.3% of Rec.) *	2030.8 ± 809.0 * (82.3% of Rec.)	2550.4 (1907.7–3165.1) * (170.0% (127.2–211.0%) of Rec.) *
Track and Field	793.9 ± 390.1 * (61.4% (44.7–104.9%) of Rec.) *	15.8 ± 4.2 (93.9% of Rec.)	162.6 (122.9–188.3) (77.7% of Rec.) *	2311.8 ± 902.6 (92.4% of Rec.)	3034.9 ± 1402.6 * (202.3% of Rec.) *
Soccer	713.2 (500.5–904.9) (63.2% (42.1–83.2%) of Rec.) *	12.0 (10.7–17.1) (76.1% (60.6–99.9%) of Rec.) *	243.0 (167.8–303.4) (71.9% (53.2–94.8%) of Rec.) *	2200.0 (1868.4–2988.2) (98.3% of Rec.)	2678.3 ± 899.6 * (178.6% of Rec.) *
Golf	559.0 ± 126.9 * (51.5% of Rec.) *	11.1 ± 3.5 * (65.4% of Rec.) *	162.6 (122.9–188.3) (50.3% (38.1–59.6%) of Rec.)	1769.3 ± 413.6 * (70.7% of Rec.) *	2394.3 ± 639.5 * (159.6% of Rec.) *
Tennis	677.0 ± 435.4 * (64.5% of Rec.)	12.2 ± 3.1 * (69.9% of Rec.) *	186.3 ± 72.1 * (58.6% of Rec.) *	1926.5 ± 849.7 * (76.0% of Rec.)	3071.7 ± 1084.0 * (204.8% of Rec.) *

Note: Data are mean values ± SD or median values with 25% and 75% interquartile ranges; * denotes significant difference between intake and Dietary Reference Intake (DRI) recommendation.

## Data Availability

The data presented in this study are available on request from the corresponding author due to participant privacy reasons.
